# Fine needle aspiration cytology including the analysis of human papilloma virus (HPV) DNA enhances the diagnostic workup of solitary cystic neck lesions in a population with a high incidence of HPV positive oropharyngeal cancer

**DOI:** 10.2340/1651-226X.2025.42078

**Published:** 2025-02-17

**Authors:** Evelina Jörtsö, Linda Marklund, Martin Harper Hysek, Anders Näsman, Lalle Hammarstedt-Nordenvall, Mattias von Beckerath, Tina Dalianis, Rusana Bark

**Affiliations:** a Division of Ear Nose and Throat Diseases, Department of Clinical Sciences Intervention and Technology, Karolinska Institutet, Stockholm, Sweden; b Medical Unit Otorhinolaryngology, Karolinska University Hospital, Stockholm, Sweden; c Department of Surgical Sciences, Section of Otolaryngology and Head and Neck Surgery, Uppsala University, Uppsala, Sweden; d Department of Oncology-Pathology, Karolinska Institutet, Stockholm, Sweden; e Department of Clinical Pathology, Karolinska University Hospital, Stockholm, Sweden; f Medical Unit Head, Neck, Lung, and Skin Cancer, Theme Cancer, Karolinska University Hospital, Stockholm, Sweden

**Keywords:** FNAC, branchial cleft cyst, cystic metastasis, tonsillar cancer, base of tongue cancer, cancer of unknown primary, cervical cancer

## Abstract

**Background and purpose:**

Distinguishing branchial cleft cysts (BrCCs) from cystic metastases of human papillomavirus (HPV) positive tonsillar or base of tongue squamous cell carcinoma and cancer of unknown primary (CUP) is challenging. Fine needle aspiration cytology (FNAC) from cystic metastasis can be nonrepresentative, while reactive squamous cells from BrCC can be atypical. It is unclear whether benign characteristics and the absence of HPV-DNA in FNAC can enhance distinguishing BrCC from a cystic metastasis; therefore, we investigated here.

**Patients/materials and methods:**

Morphology and HPV-DNA in FNAC were reevaluated preoperatively and correlated to final diagnosis of 304 BrCC and CUP patients at Karolinska University Hospital during 2016-2023.

**Results and interpretation:**

All 176 cases finally diagnosed as BrCC were HPV-DNA negative in the preoperative FNAC. HPV-DNA was present in 100/128 (78.1%) of the FNAC with a solitary cystic neck metastasis and in 3/3 CUPs separately investigated on surgical specimens, which is distributed in 40/58 (69.0%) CUP, 40/41 (97.6%) tonsillar cancer, 21/22 (95.5%) base of tongue cancer, 2/2 uterine cervical cancer, and 0/5 non-HPV-related cancers.

**Interpretation:**

All cases with final BrCC diagnosis were HPV-DNA negative in FNAC. HPV-DNA was only present in FNAC of malignant cystic neck masses of HPV-related tumors or CUP. The data suggest that HPV-DNA analysis in FNAC enhances the diagnostics of cystic masses of the neck. A FNAC with a benign morphology and the absence of HPV-DNA indicated a BrCC, while an HPV-DNA positive aspirate irrespective of morphology suggested an HPV-DNA positive cancer or CUP.

## Introduction

Solitary cystic masses occurring in the neck can encompass a spectrum from benign entities like branchial cleft cysts (BrCCs) to malignant conditions, including cystic metastases stemming from oropharyngeal squamous cell carcinoma (OPSCC), cancer of unknown primary (CUP), as well as papillary thyroid cancer (PTC) [1, 2]. BrCCs typically arise from congenital epithelial cysts, primarily due to the failure of second branchial cleft obliteration [3]*.* BrCC presents as a lateral neck mass typically situated anterior to the sternocleidomastoid muscle in the mid part of the neck. Head and neck squamous cell carcinoma (HNSCC) often present with a node metastasis in the neck, and in many cases, the metastasis is solid [4].

For human papilloma virus (HPV)-related HNSCC, typically OPSCC and CUP, the metastasis in the neck is often cystic, and because of the shared anatomical location, differentiation between BrCC, OPSCC, and CUPs poses a diagnostic challenge, especially in patients over 40 years of age [2, 4–6]*.* Notably, in studies involving surgically excised BrCCs, the prevalence of an unsuspected HNSCC metastasis ranged from 3.6% to 9.2%, with a higher incidence (23.5%) in patients over 40 years of age, and for a PTC metastasis, the prevalence was up to 7.1% [5, 7–11].

In many Western countries, most OPSCC cases – with dominance of tonsillar and base of tongue squamous cell carcinoma (TSCC and BOTSCC) – are HPV-DNA positive, and the latter is also the case for most CUP of the head and neck region [12–17]. Moreover, it is known that most of the cystic neck metastasis from OPSCC and CUP is HPV-DNA positive, and that the detection of HPV-DNA in the fine needle aspiration cytology (FNAC) is correlated to the presence of HPV-DNA positive OPSCC or CUP [18–22]*.* However, it is not sufficiently investigated whether HPV-DNA is present in FNAC samples of BrCC, and if the use of FNAC with HPV-DNA analysis in the preoperative protocol can be used to distinguish BrCC from cystic metastasis.

At the Karolinska University Hospital, Stockholm, Sweden, the diagnostic guidelines for BrCC evaluation vary by age. Patients 40 years or older are subjected to a more extensive and invasive workup resembling that for CUP, including computed tomography (CT) and magnetic resonance imaging (MRI), followed by panendoscopy with bilateral tonsillectomy and biopsies of the base of tongue and nasopharynx to minimize the risk of missing a potential primary tumor [23]. Consequently, this workup includes additional surgery causing pain and suffering for the patients.

Even though FNAC has a high accuracy (>90%) for the diagnosis of cervical solid masses, distinguishing between benign and malignant squamous cells in aspirates of cystic lesions can render false-negative rates as high as 32–63% [24, 25]. In previous studies with a limited number of patients, our group has found no presence of HPV or p16 in patients with a final histopathological diagnosis of BrCC, while HPV and/or p16 was only present in HPV-DNA positive OPSCC [23, 26].

In this study, we aimed to investigate whether FNAC morphology combined with HPV-DNA analysis in the aspirate from a solitary cystic neck lump could be used to distinguish a cystic metastasis from a BrCC and spare patients >40 years of age with a FNAC presenting a benign morphology and the absence of HPV-DNA the extensive diagnostic workup.

## Patients/material and methods

### Study cohort

**Figure 1:**
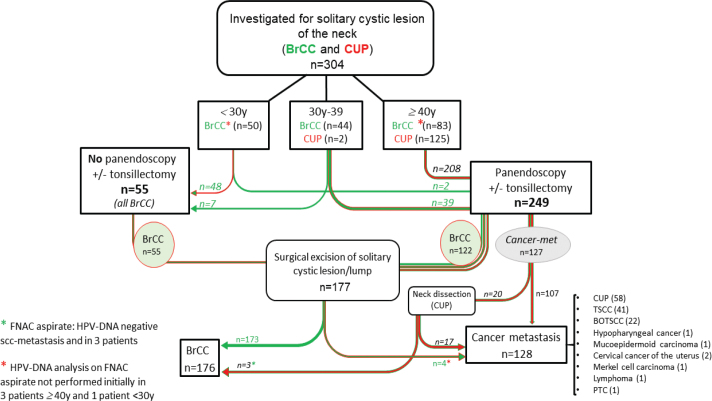
A flow chart representing the presented study, with 304 patients investigated with a solitary cystic lesion of the neck. Abbreviations: BrCC: Branchial cleft cyst; CUP: Cancer of unknown primary; FNAC: Fine needle aspirate cytology; HPV: human papillomavirus; TSCC: tonsillar squamous cell carcinoma; BOTSCC: base of tongue squamous cell carcinoma; PTC: primary thyroid cancer; SCC: squamous cell carcinoma.

All patients who had undergone diagnostic workup for suspected BrCC or CUP at Karolinska University Hospital, Stockholm, Sweden, between 2016 and 2023 were included in this study. Using the NOMESCO surgical procedure codes ENB40, UEN02, 05, 12, 15, UDH02, 05, UJC02, and ENB10, in total, 557 patients were identified. After reviewing the medical records, 253/557 patients were excluded: 71 were <18 years of age, and 182 were investigated for other diagnoses than BrCC or CUP (Supplementary Table 1). Of the remaining 304 patients included in the study, 177 were initially and preoperatively investigated under the preliminary diagnosis of BrCC, while 127 were initially and preoperatively investigated for the preliminary diagnosis of CUP. Patients’ characteristics are shown in Table 1, and a flow chart of the present study is presented in Figure 1 and in more detail later.

**Table 1:** Demographic data in 304 patients undergoing a fine needle aspirate due to a cystic neck mass.

Total cohort^1^	

Patients	304
Female	128 (42.1%)^4^
Male	176 (57.9%)^4^
Mean age, years (range)	50.3 (19–95)
Median age, years	52
Smoking (former or current)	136 (44.7%)

Investigated for BrCC^2^	

Patients	177 (58.2%)
Female	86 (48.6%)^4^
Male	91 (51.4%)^4^
Mean age, years (range)	40.7 (18–79)
Median age, years	37
Smoking (former or current)	66 (37.3%)

Investigated for CUP^3^	

Patients	127 (41.8%)
Female	42 (33.1%)^4^
Male	85 (66.9%)^4^
Mean age, years (range)	63.7 (36–95)
Median age, years	63
Smoking (former or current)	70 (55.1%)

1 Number of patients,

2 Branchial cleft cyst,

3 Cancer of unknown primary of the head and neck region,

4 Percentage of corresponding cohort.

### Diagnostic workup

The investigation protocol for solitary cystic neck masses at Karolinska University Hospital, Stockholm, Sweden, differs depending on the patient’s age [23]. For patients <30 years of age, the protocol typically involves FNAC morphology, including HPV-DNA analysis of the FNAC material. For patients 30–39 years of age, please see Figure 1. Patients with >40 years of age are subjected to a FNAC, including morphology and an HPV-DNA analysis, as well as a CT and MRI, panendoscopy with biopsies of the nasopharynx and the base of tongue, and a bilateral tonsillectomy (TE). When all the data from the aforementioned investigations are benign and the FNAC has benign morphology and is found to be HPV-DNA negative, then the cyst will be removed. If any of the investigations suggested a malignancy, or the FNAC was HPV-DNA positive, a detailed therapeutic handling was performed, subsequent to further assessment of additional diagnostics at a multidisciplinary conference.

Data were initially available for the presence of HPV-DNA in 264/304 of the included cases. In the 40 cases, where the HPV-DNA analysis in FNAC was missing/not carried out during the diagnostic workup, a reevaluation of the preoperative cytology and an HPV-DNA analysis on the cyst aspirates were possible to perform in 36/40 cases. This material was obtained by scraping from glasses as this was the only material saved in the archive. The primary criteria for suitability were that enough materials, for example, at least one additional representative, smear remained in the archive for possible reevaluation if needed at a later point. In 3/40 remaining cases (all CUP), this was not possible due to lack of materials, and the HPV-DNA analysis was instead completed in available tissue from the surgical resection; in 1/40 cases, this was not done due to the diagnosis of PTC.

This study has been approved by the Ethical Committee (ethical review numbers: 2005/431-31/4; 2005/1330-32; 2009/1278-31/4; 2010/1117-32; 2015/0157-32; 2023-05249-01).

### HPV-DNA analysis

HPV-DNA analysis was done at the Dept. of Clinical Pathology, Karolinska University Hospital, using the HPV assay ‘BD Onclarity™ HPV Assay’ (BD, USA) on frozen pellets of needle washings with Phosphate Buffered Saline (PBS) solution (Merck, Germany). If frozen pellets were not available, a May-Grünwald-Giemsa-stained or a Papanicolaou-stained microscopic slide with the representative material was selected for scrapings, and this material was used for analysis.

### Statistics

Our study consisted of two groups, that is, those investigated for either BrCC or CUP. Comparisons were made regarding morphology and the HPV-DNA analysis obtained from the FNAC, as well as the final diagnosis. Sensitivity, specificity as well as negative and positive predictive values were calculated for detecting a cystic metastasis with FNAC only and compared to FNAC including an HPV-DNA analysis. The four cases with missing HPV-DNA data in the FNAC upon initial reevaluation were excluded from the analyses regarding HPV-DNA data alone and together with FNAC morphology.

## Results

### Presence or absence of HPV-DNA in FNAC material as well as in a limited number of surgical samples in relation to the initial and final diagnoses

The analysis of HPV-DNA was subsequently completed in material from 300 FNAC and was found in FNAC in 100/304 (32.9%) and in 3/3 (100%) surgical samples, of which the latter three were ultimately diagnosed as CUP. HPV was thereby found in 103/304 (33.9%) of the patients when including data from the final diagnostic modalities (Table 2). Of note in the one initial BrCC, the first FNAC was HPV-DNA positive but with a very low signal. The repeated FNAC was HPV-DNA negative, and due to being >40 years, this patient was investigated as having a metastasis but was later shown to have a BrCC. Details of all the patients’ demographics and ages are shown in Table 1.

**Table 2:** Final diagnosis in relation to human papilloma virus (HPV) and HPV types.

Final histopathological diagnosis	HPV results								
	HPV negative	HPV positive	Types of HPV (in FNAC)					Types of HPV (in surgical specimen)	
			*HPV 16*	*HPV 18*	*HPV 33*	*HPV 45*	*HPV n.d.*	*HPV 16*	*HPV 33*
BrCC (*n* = 176)	176 (100%)	0	-	-	-	-	-	-	-
Cystic metastasis (*n* = 128)	24	103 (80.5%)	-	-	-	-	1	-	-
CUP (*n* = 58)	18	40* (69.0%)	31		6	-	-	2*	1*
TSCC (*n* = 41)	1	40^α^ (97.6%)	38	1	1	-	-	-	-
BOTSCC (*n* = 22)	1	21^β^ (95.5%)	19		2	-	-	-	-
Hypopharyngeal cancer (*n* = 1)	1	-	-	-	-	-	-	-	-
Mucoepidermoid cancer (*n* = 1)	1	-	-	-	-	-	-	-	-
Cervical cancer of the uterus ( *n* = 2)		2	-	-	-	2	-	-	-
Merkel cell cancer (*n* = 1)	1	-	-	-	-	-	-	-	-
Lymphoma (*n* = 1)	1	-	-	-	-	-	-	-	-
PTC (*n* = 1)	-	-	-	-	-	-	1	-	-

BrCC: Branchial cleft cyst; CUP: cancer of unknown primary of the head and neck region; TSCC: tonsillar squamous cell carcinoma; BOTSCC: base of tongue squamous cell carcinoma; PTC: papillary thyroid cancer; n.d.: not done.

* In three patients, HPV analysis was not done (n.d.) due to lack of material in FNAC, but they were later determined as HPV-DNA positive in the histopathology of the excised cystic metastasis (two had HPV16 and one had HPV33).

α Six patients with contralateral TSCC and one with synchronous bilateral TSCC.

β One patient presented with synchronous BOTSCC and TSCC; in this study, it is counted as BOTSCC because of tumor size.

Among the initial 177 suspected BrCC cases, four cases were upon reevaluation subsequentially diagnosed as a malignancy, of which three were diagnosed as HPV-positive CUP, while one was a PTC (Table 3). Among the initial 127 suspected CUP cases, three were subsequentially diagnosed as BrCC, all three were HPV-DNA negative in FNAC as shown in Table 3 and the flow chart of Figure 1. This rendered a final diagnosis of 176 BrCC and 128 cases of cystic metastasis.

**Table 3:** Descriptive outcome according to the primary investigation in Branchial cleft cysts (BrCCs) and Cancer of Unknown Primary (CUP).

Final diagnosis	Investigated for	
	CUP (*n* = 127)	BrCC (*n* = 177)
Cystic metastasis (*n* = 128)	124	4^1^
BrCC (*n* = 176)	3^2^	173

1 Three CUP and one primary thyroid cancer (PTC);

2 Three patients with initially malignant/unclear morphology in FNAC and HPV-DNA negative in aspirate, and after surgical excision had benign histopathology.

More specifically, HPV-DNA was not found in any of the 176 cases with a final BrCC diagnosis but present in 100/128 (78.1%) of the FNAC, and when including data from the three surgical CUP samples, in total, 103/128 (80.5%) cases of cystic metastasis (Table 2).

Upon final diagnosis, HPV-DNA was thereby present in the specimens of 40/58 (69.0%) patients with CUP, 40/41 (97.6%) patients with TSCC, 21/22 (95.5%) patients with BOTSCC, and 2/2 (100%) patients with cervical cancer of the uterus (CC) (Table 2).

Among cystic metastases (*n* = 128), we observed the following HPV types: HPV16 dominated with 90/128 (70.3%) of the cases, followed by HPV33 in 10/128 (7.8%), HPV45 in 2/128 (1.6%), and HPV18 in 1/128 (0.8%) of the cases. All of these were from patients with an HPV-related cancer as the primary tumor origin, that is, either CUP, TSCC, BOTSCC, or CC (Table 2).

HPV-DNA was thereby not present in FNAC of 18/58 (31.0%) CUP, 1/41 (2.4%) TSCC, 1/22 (4.5%) BOTSCC, 0/2 (0%) CC, and 5/5 (100%) other cancers (Table 2).

### Presence or absence of HPV-DNA in FNAC material in relation to the morphology

All patients with benign morphology and BrCC as a final diagnosis showed absence of HPV-DNA in FNAC (Table 4). The malignancy rate for patients undergoing a diagnostic workup for BrCC was 4/177 (2.3%) patients, resulting in three patients with CUP as a final diagnosis and one patient with PTC (Figure 1). Conversely, three patients from the CUP cohort initially presented with a morphologically positive squamous cell carcinoma metastasis and HPV-DNA negative FNAC, and these were later found to have a benign histopathology after neck dissection (levels I–V) with the final diagnosis of BrCC. More details regarding the final histopathology data versus the FNAC together with data on the presence or absence of HPV-DNA in the FNAC are presented in Table 4.

**Table 4:** Histopathology data versus fine needle aspirate cytology (FNAC) morphology and the presence or absence of HPV-DNA in the FNAC HPV status.

FNAC morphology	Histopathology results	
	Cystic metastases (*n* = 128)	BrCC (*n* = 176)
Malignant	117 (91.4%)	3** (1.7%)
Benign	11* (8.6%)	173 (98.3%)
HPV status in FNAC		

HPV-DNA positive	100 (78%)	0 (%)
HPV-DNA negative	24 (18.8%)	176 (100%)
HPV-DNA n.d.	4^α^ (3.1%)	0 (0%)
HPV status (in FNAC or surgical specimen)		

HPV positive	103^β^ (80.5%)	0 (0%)
HPV negative	24 (18.8%)	176 (100%)
HPV-DNA n.d.	1^α^ (0.8%)	0 (0%)
FNAC results		

HPV-DNA positive and/or malign FNAC	123 (96.1%)	3*^α^* (1.7%)
HPV-DNA negative and benign FNAC	1* (0.8%)	173 (98.3%)
HPV-DNA n.d.	4^α^ (3.1%)	0 (0%)

* Cystic metastasis with an initial benign FNAC morphology was found in 11 patients, as follows:

In 7/11 patients initially investigated for CUP with FNAC benign morphology, the presence of HPV-DNA was tested in the FNAC upfront and identified in 5/7 samples, which indicated the presence of a malignancy, with 2/5 TSCC, 2/5 CUP, and 1/5 patient with synchronous BOTSCC and TSCC. Of the remaining two patients, in the HPV-DNA analysis, one could not be performed due to lack of FNAC material, and HPV33 was disclosed in the surgical specimen. And the other with suspected malignancy in the radiological workup later showed a mucoepidermoid cancer.

In 4/11 patients initially investigated as BrCC with benign morphology in the FNAC, HPV-DNA analysis was not done during diagnostic workup but was attempted at reevaluation in the FNAC: 1/11 was HPV33 positive; 2/11 had too little material from FNAC, but when HPV-DNA analysis was assessed in the surgical specimen, these were HPV16 positive. Consequentially, 3/11 patients had HPV-DNA positive CUP, and the remaining 1/11 patient had primary thyroid cancer (PTC).

α HPV-DNA analysis in FNAC was not performed in four patients because lack of material in three patients (all CUP) and other diagnosis in one patient (PTC).

β Three with HPV found in the surgical specimen.

** BrCC with initial malignant FNAC morphology: Three patients had malignant morphology and were HPV-DNA negative in FNAC and under CUP investigation; ultimately, after neck dissection, all three were found to be benign, thus diagnosed as BrCC.

### Sensitivity, specificity, and negative and positive predictive values calculated for detecting a cystic metastasis with FNAC alone as compared to FNAC including an HPV-DNA analysis

When calculating the statistics on HPV-DNA analysis alone and together with FNAC morphology, four cases were excluded due to that an HPV-DNA analysis was not done in their FNAC due to lack of material in three cases and due to diagnosis of PTC in one. Of these, three were HPV positive in the surgical specimen and would likely have had an HPV-DNA positive FNAC if the FNAC had rendered enough material to perform the analysis, and one was PTC and would likely not been HPV-DNA positive in the FNAC.

The negative predictive value for FNAC morphology alone was 94.0%, which is less than 99.4% for FNAC morphology together with HPV-DNA analysis. The sensitivity of FNAC morphology alone was 91.4%, which was lower than the sensitivity for FNAC morphology and HPV-DNA analysis, which was 99.2%, for finding cystic metastases. The positive predictive value for HPV-DNA analysis in FNAC was 100%, while the FNAC morphology alone was 97.5%. Interpreting the data with caution, due to the low numbers of patients, adding HPV-DNA analysis to the FNAC morphology most likely slightly enhanced the prediction of individuals with benign conditions as well as enhanced the finding of cystic metastasis of patients with an HPV positive cancer.

In summary, for patients investigated for a solitary cystic neck mass, when the FNAC identified both a benign morphology and the absence of HPV-DNA in the cyst aspirate, none of the patients had a final malignant diagnosis. In contrast, the presence of HPV-DNA always indicated the presence of an HPV-related cancer.

**Figure 2:**
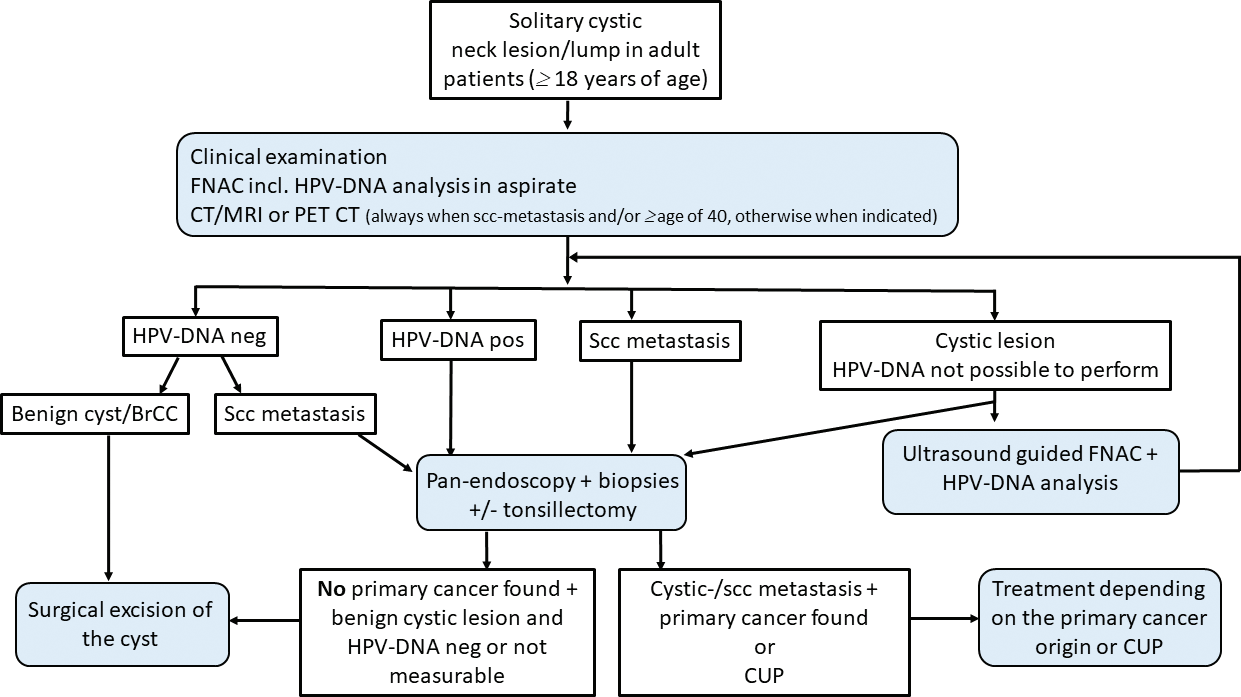
A modified investigation flow chart protocol for solitary cystic neck lesion/lump in adult patients.

A flow chart of the analysis in this study and a summary of the above data have been illustrated in Figure 1. Based on the acquired data, we suggest a new work up protocol, which is presented in Figure 2 and discussed in further detail later.

## Discussion

In this study, morphology and HPV-DNA analysis in FNAC were compared in two cohorts of patients with a similar diagnostic workup for a solitary cystic neck mass: BrCC and CUP. None of the 176 patients with a final diagnosis of BrCC had an HPV-DNA positive FNAC. In contrast, all patients with an HPV-DNA positive FNAC had a cystic metastasis derived from an HPV-positive malignancy, with the majority being OPSCC or CUP, and two having CC.

Not detecting HPV-DNA in any FNAC of the BrCC samples aligns with our previous findings that HPV is not found in congenital lateral neck cysts as well as strengthens the hypothesis that it is feasible to distinguish a cystic metastasis of HPV-positive OPSCC from BrCC by combining the presence of benign cytopathology and HPV-DNA analysis in FNAC [20, 23, 26].

Furthermore, finding HPV-DNA in cystic aspirate indicates the presence of an HPV-related tumor and can be used to direct the search for its site of origin. The incidence of HPV-DNA positive OPSCC has increased the last decades, especially in the Western world, and it is well-known that HPV-positive OPSCC tends to produce cystic metastasis, thereby posing a diagnostic dilemma for BrCC diagnosis [12, 14, 17, 27]. Although there is enough evidence in the literature supporting the use of FNAC as a valid method for gaining material for HPV testing, the absence of HPV-DNA *alone* in a cyst aspirate cannot be considered sufficient in confirming the benign nature of the BrCC. However, the presence of HPV-DNA in a FNAC certainly indicates a malignancy and strongly points to the oropharynx as the main site of origin, with consequences for subsequent clinical management and treatment decisions [18, 20, 21, 23].

Upon a detailed analysis, 128 patients in this study had a malignant morphology in their FNAC. Many, 63/128 (49.2%), of the primary tumors were located in the oropharynx, 41/128 (32.0%) TSCC and 22/128 (17.2%) BOTSCC. All but two, 61/63 (96.7%) being HPV positive. A primary tumor could not be located in 58/128 (45.3%) patients, thus diagnosed as CUP with 40/58 (69.0%) being HPV positive. It is likely that some patients with a final HPV-DNA positive CUP diagnosis may have had a very small primary tumor, possibly in the oropharynx somewhere in the Waldeyer’s lymphatic ring.

Among the patients investigated for BrCC, three patients had a diagnostic FNAC performed, where the morphology was inconclusive with no epithelial cells, and the HPV-DNA analysis was not initially made. After a negative panendoscopy including TE and biopsies, the cyst was surgically removed and found to carry a cystic metastasis. Upon reevaluation of the initial FNAC samples, HPV-DNA analysis was performed, presenting one as HPV-DNA positive (HPV33), and the other two not possible to reevaluate, but when the surgically excised cysts were assessed, one was HPV16 and one was HPV33 positive. If the flow chart (Figure 2) including an HPV-DNA analysis on the initial cyst aspirate had been followed, these patients would likely been correctly diagnosed with a malignancy before surgery.

Nevertheless, among patients investigated for CUP, the diagnostic workup resulted in an HPV-negative CUP diagnosis for 20 patients who then underwent therapeutic neck dissection as a part of CUP treatment. Of these, 3/20 did not have a cancer in the histopathology report on the neck dissection specimen and were instead diagnosed as BrCC.

There are definitely diagnostic challenges, and several studies show a tendency for cystic metastasis to occur more frequently in patients >40 years of age [1, 2, 28]. Moreover, it is well-known that distinguishing a well-differentiated cystic squamous cell carcinoma metastasis from a benign condition by radiological and cytological investigation is demanding. FNAC has a diagnostic morphological accuracy of >90% for solid masses, while the technique can be less reliable in cystic lesions, so distinguishing between BrCC and cystic squamous cell carcinoma metastasis is intricate [3]. This leads to patients >40 years of age with a suspected BrCC have to undergo an extensive diagnostic workup including a panendoscopy with bilateral TE and biopsies of the nasopharynx and from the base of tongue, before surgical excision of the cyst. This is analogous to the diagnostic workup for head and neck CUP recommended by the ASCO guidelines, thereby including a complete upper aerodigestive tract evaluation of mucosa including directed biopsies and ipsilateral TE [1]. Notably, in the cases we had followed the ASCO guidelines, seven cases of TSCC had been missed: since six patients presented with contralateral cystic metastasis, and one had bilateral TSCC.

Cystic metastases in the neck in individuals <40 years of age are relatively uncommon, but they can occur, particularly in association with certain types of cancers, such as PTC, OPSCC, nasopharyngeal cancer, lymphoma, lung cancer, and skin cancer of the head [5, 7–11, 29]. In our cohort, we only had one PTC case in the group investigated for suspected BrCC. This patient was 26 years old and presented with a cystic neck lesion in neck level IV, which is an unusual location for BrCC. Also, only one FNAC attempt was made with an inconclusive result. If the FNAC had been repeated and combined with an ultrasound of the thyroid and the central lymphatic compartment, as well as HPV-DNA, TTF-1, or a thyroglobulin analysis on the cyst aspirate, the chances of making a correct initial diagnosis had likely increased.

In summary, none of the patients with both an HPV-DNA negative FNAC and a conclusive benign morphology had a malignancy in the surgically removed BrCC, while all patients with an HPV-DNA positive FNAC had an HPV-related malignancy. The data presented here suggest that the extensive diagnostic workup for the investigation of solitary cystic neck mass may not be necessary if the FNAC has a benign morphology and presents a negative HPV-DNA-analysis taken from a cyst aspirate, while the opposite should be the case for those with an HPV-DNA positive result [23].

As FNAC is already part of the diagnostic workup of BrCC, we suggest that the HPV-DNA-status of the FNAC should be performed as a part of the investigation for BrCC, as illustrated in Figure 2, demonstrating an improved flow chart protocol for the investigation of a solitary neck cyst, such as BrCC. This could reduce the morbidity, including postoperative pain, hemorrhage, etc. for a considerable number of patients and, at the same time, limit the use of some of the available hospital resources. However, if the initial FNAC or an ultrasound-guided FNAC analysis from the cyst aspirate exhibits either a malignant morphology and/or positive HPV-DNA or inconclusive data, then an extensive diagnostic workup should be performed for a suspected malignancy, regardless of age (Figure 2). This flow chart further highlights the usefulness of HPV-DNA-testing in the cystic aspirate (in order not to miss a possible HPV-positive OPSCC or CUP) and also in cystic lesions with benign cytological morphology.

There are limitations in this study. One is the retrospective setting and another that it is a single center study with a limited number of patients. However, the Swedish healthcare system provides that all patients investigated for CUP and BrCC in the Stockholm region are subjected to the same diagnostic workup, including FNAC and HPV-DNA analysis. Another limitation could be that the proportion of HPV-positive OPSCC is high in Sweden, which would enhance our possibilities to use the analysis of HPV-DNA as compared to other geographic areas with a much lower proportion of HPV-positive OPSCC. Finally, statistically little difference was observed upon adding HPV-DNA analysis to the FNAC, but one should keep in mind that the high numbers of FNAC accuracy are true for the setting in the county of Stockholm, where there are well experienced cytologists working full-time performing FNAC. In a region with a smaller population and less-experienced cytologists/ENT doctors performing the FNAC, the accuracy yield of FNAC data could be lower.

## Conclusion

All patients with a final diagnosis of BrCC had an FNAC presenting a benign morphology that was HPV-DNA negative, suggesting that the addition of HPV-DNA analysis in FNAC enhances the possibility of distinguishing between BrCC and cystic metastases of HPV-related cancer. The analysis of HPV-DNA has a definite role in the diagnostics of cystic neck masses and should be implemented in clinical practice.

We suggest a new protocol for investigating solitary cervical neck cysts when the diagnostic FNAC shows benign morphology and is HPV-DNA negative and recommend surgical excision of the solitary cystic mass, without previous bilateral TE and blind biopsies of the base of tongue and the nasopharynx, regardless of the patients’ age, thus reducing morbidity. Should HPV-DNA be found in the FNAC of the cystic mass, then a further investigation under the suspected diagnosis of HPV-related cancer should be done regardless of the morphology and the age of the patient.

## Supplementary Material

Fine needle aspiration cytology including the analysis of human papilloma virus (HPV) DNA enhances the diagnostic workup of solitary cystic neck lesions in a population with a high incidence of HPV positive oropharyngeal cancer

## Data Availability

Data supporting this study are included within the articles and in references [21, 23], and further data are available upon request.
