# Supplementary material for: Fine needle aspiration cytology including the analysis of human papilloma virus (HPV) DNA enhances the diagnostic workup of solitary cystic neck lesions in a population with a high incidence of HPV positive oropharyngeal cancer
Source: Acta Oncol. 2025 Feb 17;64:42078. doi: 10.2340/1651-226X.2025.42078 (PMC11848946; doi:10.2340/1651-226X.2025.42078)
Supplement: Fine needle aspiration cytology including the analysis of human papilloma virus (HPV) DNA enhances the diagnostic workup of solitary cystic neck lesions in a population with a high incidence of HPV positive oropharyngeal cancer [file AO-64-42078-s1.pdf]

Supplementary material has been published as submitted. It has not been copyedited, or typeset by Acta Oncologica

Supplementary Table 1. Patients initially investigated for a branchial cleft cyst or a Cancer of Unknown Primary of the head and neck region during 2016-2023 and excluded from the present study.

Excluded (n=182)[1]			
**Investigated for BrCC[2] (n=39)**		**Investigated for CUP[3] (n=143)**	
Neck fistula	6	Surgery cancelled because non-palpable at time of surgery	1
Other fistula	2	Lymphatic malformation	1
Laryngeal cyst	2	FNAC[4] showed suspected Warthin tumor in submandibular gland, patient moved – investigation incomplete	1
Benign parotid gland tumor	2	Panendoscopy + biopsies for known or suspected primary cancer	140
Thymus cyst	1		
UPPP	1		
Lymph node	4		
Lipoma	2		
Dermoid cyst	3		
Schwannoma	1		
Thyreoglossal neck cyst	4		
Myofibroma	1		
Subcutaneous cyst	2		
Atheroma	1		
Rhabdofibroma/myofibroma	2		
Lymphoepithelial cyst	5		

[1]Initially, 557 patients were identified using the N the NOMESCO surgical procedure code ENB40 (for removal of the cystic lesion/BrCC), UEN02, 05, 12, 15 (Hypopaharyngoscopy- and oropharyngoscopy with or without biopsy), UDH02, 05 (rhinopharyngoscopy with or without biopsy), UJC02 (rigid esophagoscopy) and ENB10 (tonsillectomy). Of these 182 were excluded and their details are presented in the Table above.

[2]Branchial cleft cyst, [3]Cancer of unknown primary of the head and neck region, [4]Fine needle aspirate cytology (FNAC)
